# The Roles of Exosomes in Immunoregulation and Autoimmune Thyroid Diseases

**DOI:** 10.3389/fimmu.2021.757674

**Published:** 2021-11-15

**Authors:** Junli Zou, Huiyong Peng, Yingzhao Liu

**Affiliations:** ^1^ Department of Endocrinology, The Affiliated People’s Hospital of Jiangsu University, Zhenjiang Medical School of Nanjing Medical University, Zhenjiang, China; ^2^ Department of Laboratory Medicine, The Affiliated People’s Hospital of Jiangsu University, Zhenjiang Medical School of Nanjing Medical University, Zhenjiang, China; ^3^ Department of Genetic Toxicology, The Key Laboratory of Modern Toxicology of Ministry of Education, Center for Global Health, School of Public Health, Nanjing Medical University, Nanjing, China

**Keywords:** exosomes, immune regulation, Hashimoto’s thyroiditis, Graves’ disease, autoimmune thyroid diseases (AITD)

## Abstract

Exosomes are extracellular microvesicles (30-150 nm) released from cells that contain proteins, lipids, RNA and DNA. They can deliver bioactive molecules and serve as carriers facilitating cell-cell communication, such as antigen presentation, inflammatory activation, autoimmune diseases (AIDs) and tumor metastasis. Recently, much attention has been attracted to the biology and functions of exosomes in immune regulation and AIDs, including autoimmune thyroid diseases (AITDs). Some studies have shown that exosomes are involved in the occurrence and development of AITDs, but they are still in the preliminary stage of exploration. This review mainly introduces the association of exosomes with immune regulation and emphasizes the potential role of exosomes in AITDs, aiming to provide new research strategies and directions for the pathogenesis and early diagnosis of AITDs.

## Introduction

Autoimmune thyroid diseases(AITDs) are thyroid diseases caused by autoimmune disorders, mainly including Hashimoto’s thyroiditis (HT) and Graves’ disease (GD) (1). This disease is mainly manifested by infiltration of lymphocytes and the production of autoantibodies, among which autoantibodies interfere with thyroid function, leading to hypothyroidism or hyperthyroidism (2). AITDs are usually accompanied by the production of thyroid peroxidase antibodies (TPOAb), thyroglobulin antibodies (TGAb), thyrotropin receptor antibodies (TRAb) and other autoantibodies (3). The pathogenesis of this disease is very complicated and is mainly classified into genetic predisposition, environmental implications, and immunological factors which are particularly important (4). T lymphocytes and their secreted cytokines play an indispensable role in the regulation of immune response (5). Dysfunction of these T cells or abnormal expression of these cytokines can lead to the destruction of immune tolerance and abnormal immune responses during the development of AITDs. In recent years, the incidence of AITDs has been on the rise, causing adverse effects on people’s quality of life, and increasing the risk of some non-thyroid diseases, such as other AIDs (type 1 diabetes, systemic lupus erythematosus, etc.), cardiovascular diseases, thyroid cancer, etc. (6). Therefore, it is of great importance to search for early diagnostic markers and effective therapeutic targets of ATIDs.

Recently, the finding that exosomes are involved in AIDs has been a major breakthrough in the field, unveiling their capacity to modulate immune responses. Exosomes are nanoscale extracellular vesicles secreted by various cells that carry specific substances such as proteins, lipids and nucleic acids to conduct cell-to-cell signal transduction, participate in the occurrence of a variety of diseases and may become new biomarkers (7). In AIDs, antigen-presenting cells (APCs) presenting autoantigens can spread MHC (major histocompatibility complex)/polypeptide complexes to secondary lymph nodes by releasing exosomes, indirectly activating more antigen-specific T cells and aggravating the occurrence and development of AIDs (8). As one of the most prevalent autoimmune diseases, AITDs are caused by an abnormal autoantigen clearance system and an imbalance in immunoregulation and inflammatory mechanism (9). Autoimmune intolerance is one of the leading theories proposed for thyrocyte destruction in AITDs *via* the imbalance of regulatory T cells (Tregs) and T helper 17 (Th17) cells in adaptive immunity (10). Exosomes derived under pathological conditions can influence Treg and Th17 cell balance in the disease microenvironment, which may contribute to the disruption of autoimmune tolerance in AITDs. In this regard, it has been reported that (11) plasma microvesicles can regulate the differentiation of Tregs and Thl7 cells in AITDs, which is related to their cargos such as miRNAs. Therefore, exosomes and their cargos might play a role in immunoregulation and AITD pathogenesis. In this review, we will highlight the current understanding of exosomes in immunoregulation and AITDs and discuss how exosomes may contribute to AITD pathogenesis.

## The Biogenesis and Functions of Exosomes

Exosomes are extracellular vesicles synthesized and secreted by eukaryotic cells and were originally discovered in the supernatant of sheep red blood cells (12). Under a transmission electron microscope, exosomes are 30-150 nanometers in diameter (13), surrounded by bilayer phospholipid molecules (14), in the shape of plates or cups (15), and usually exist in the sucrose density layer of 1.13-1.19 g/ml (16). The biogenesis of exosomes starts within the endosomal system and occurs *via* intracellular cytoplasmic transport pathways involved in multivesicular cell fusion (17). Mathivanan et al. (18) reported the mechanism of exosome formation whereby exosomes originate from early endosomes. First, after endocytosing exogenous substances, cells combine with cytoplasmic proteins, nucleic acids and lipids to form early endosomes, which further mature into multivesicular bodies (MVBs) by a series of intracellular interactions. This is followed by fusion with the cell membrane and the release of intraluminal vesicles (ILVs) out of cells as exosomes (19, 20). Exosomes contain multifarious cargos such as proteins, lipids, DNA, messenger RNA (mRNA), and noncoding RNA and other biomolecules (21). Although exosomes may have similar surface proteins, such as membrane transport proteins, integrins, heat shock proteins (HSP60, HSP70, HSP90) and transmembrane four-protein superfamily (CD9, CD63, CD81, CD82), there is currently no widely accepted specific marker to distinguish exosomes from different cell populations (22). Due to the lipid bilayer structure, exosomes can stably exist in a variety of biological liquids without being degraded and can transfer biologically active substances into recipient cells to mediate the information exchange between cells, playing a key role in multiple diseases (23, 24).

Most cell types can actively secrete exosomes (25), such as tumor cells, endothelial cells, immune cells, stem cells, and nerve cells. Moreover, exosomes exist in different body fluids (26), including blood, cerebrospinal fluid, urine, lymph, ascites, bile, saliva, tears, amniotic fluid and breast milk. Exosomes have a wide range of sources and various functions, which are closely related to the cellular origin of exosomes. To date, overwhelming evidence indicates that exosomes from different cells have different biological characteristics and will evoke totally different responses in recipient cells under the influence of their contents, tissue microenvironment, receptor cells and other factors. For example, tumor cell-derived exosomes can participate in tumor metastasis, promote or inhibit tumor progression, and promote angiogenesis (27). Exosomes derived from bone cells have the functions of bone remodeling, bone metabolism, osteoblast and osteoclast differentiation (28). Exosomes derived from mesenchymal stem cells (MSCs) are able to facilitate immune regulation, angiogenesis promotion, information transmission, tissue repair and cell proliferation (29). Exosomes, serving as circulating biomarkers, play pivotal roles in intercellular communication under physiological and pathological conditions, such as immune signal transduction, inflammation, angiogenesis, and tissue repair (30–32). Due to their inherent properties, such as stability, biocompatibility, and invisibility, exosomes have also emerged as promising therapeutic delivery tools. Several studies have investigated the therapeutic potential of exosomes in immunoregulation, revealing that exosomes containing anti-inflammatory molecules can be used as immunomodulators for the treatment of inflammation, hypersensitivity and autoimmune diseases (33). Hence, we believe there is tremendous future potential for the use of exosomes in therapeutic and diagnostic area of diseases and many other applications.

## The Roles of Exosomes in the Immune System

Exosomes are rich in many bioactive molecules (chemokines, inflammatory factors, signal transduction factors, HSPs, cell specific antigens, various RNAs, etc.) or they carry proteins with special functions (adhesion molecules, costimulatory molecules, ligands, receptors, etc.) on their surface. Notably, exosomes contain specific noncoding RNAs, such as microRNAs (miRNAs) and circular RNAs (circRNAs) that can be functionally transferred to target cells, consequently leading to immune activation and suppression (34). Similarly, Okoye et al. (35) found that Treg-derived exosomes can transport miRNAs (such as Let-7d) to Th1 cells, leading to immunosuppression and the prevention of systemic disease. Exosomes modulate the immune response mainly through two mechanisms: exosomes directly act on target cells to activate downstream signals and exosomes regulate immunoreactions through exosomal miRNA mediation (36). The former act in three main ways: the direct action of surface signaling molecules, intracellular regulation of signaling molecules during membrane fusion, and extracellular release of bioactive components (37). Current studies have shown that exosomes secreted from tumor cells, stem cells and some exogenously stimulated immune cells can stimulate immune cells and regulate the function of the immune system (**Table 1**).

**Table 1:** Exosomes derived from various cells and their immune roles.

Source of exosomes	Exosome cargo	Functions	References
B cells	MHC, CD86, CD54	Present antigens and activate CD4^+^T cells	(38)
	C3	Promote T cells proliferation	(39)
LCL	MHC II, FasL	Promotes CD4^+^T cells apoptosis	(40)
T cells	DNA	Induce DCs	(41)
Activated T cells	FasL, TRAIL	Eliminate the activation of T cells	(42)
Tregs	Let-7i	Block IGF1R and TGF-βR1 pathways	(43)
	miR-548a-3p	Interfere with TLR4/NF-κB pathways	(44)
	miRNAs	Inhibit Th1 cells proliferation	(45)
DCs	HLA-II	Present antigens	(46)
	IL-1, NKG2D	Induce NK cells activation	(46)
Mature DCs	MHC, CD86, CD40	Promotes T cells activation and proliferation	(47)
Immature DCs	CD95L	Reduce T cells immune response	(48)
Macrophages	LPS	Induce DCs maturation	(49)
	miRNAs	Enhance inflammatory response	(50, 51)
NK cells	Fas, CD56, NKG2D	Activate immune effector cells and cytotoxicity	(21)
MSCs	PDL1, TGF-β	Inhibit T cells immune response	(52)
	IL-10, TNF-α, IFN-γ	Inhibit B cells proliferation	(53)
	miR-146a	Reduce inflammatory response	(54)
MDSCs		Inhibit T cell proliferation and promote Tregs amplification	(55)

LCL, lymphoblastoid cell line; FasL, human apoptosis-related factor ligand; DCs, dendritic cells; TRAIL, tumor necrosis factor-related apoptosis-inducing ligand; IGF1R, insulin-like growth factor 1 receptor; TGF-β1R, transforming growth factor -beta 1 receptor; TLR4, Toll-like receptor 4; NF-κB, nuclear factor κB; HLA, human leukocyte antigen; NK cells, natural killer cells; NKG2D, natural killer Group 2 member D; LPS, lipopolysaccharide; PDL1, programmed death ligand 1; IL-10, interleukin-10; TNF-α, tumor necrosis factor alpha; IFN-γ, interferon gamma; MDSCs, myeloid-derived suppressor cells.

At the same time, different immune cells also coordinate the immune process through exosomes (52). Exosomes from various immune cells transfer important information between immune cells and immune cells, as well as between immune cells and target proteins, and regulate innate and adaptive immunity by participating in antigen presentation or transporting substances such as killer proteins and inflammatory factors (53). Cells involved in innate immunity, such as macrophages, DCs, NK cells, and granulocytes, can recognize antigens through a class of pattern recognition receptors (PRRs), thus generating immune responses. Exosomes can affect the polarization of macrophages (54), the regulation and expression of DCs (55), and the killing effect of NK cells (56, 57). Adaptive immunity refers to the immune process in which T and B cells are activated, proliferate and differentiate into effector cells after receiving antigen stimulation, resulting in a series of biological effects. In adaptive immune responses, exosomes can disseminate antigens or MHC peptide complexes to increase the number of dendritic cells presenting them, or to directly interact with memory T cells (58).

The immunoregulatory effects of exosomes mainly include affecting antigen presentation, T cell activation, immunosuppression, the inflammatory response and intercellular communication (59). Tan et al. (60) summarized the antigen-presenting function of exosomes derived from APCs and the immunomodulatory functions of T cell-derived exosomes. Exosomes secreted by professional APCs, such as B lymphocytes and DCs, contain MHC I and MHC II complexes and costimulatory molecules. These exosomes have antigenic peptides on the surface, which can play a considerable role in immune regulation (61, 62). Recent studies have demonstrated that exosomes are involved in proinflammatory responses and can promote immune responses. Exosomes derived from bacteria- infected macrophages have been shown to have immunomodulatory effects and stimulate macrophages and neutrophils to secrete proinflammatory mediators, including TNF-α and RANTES (upregulating iNOS expression) (63, 64). Moreover, exosomes derived from activated CD8^+^ T cells have been demonstrated to express bioactive FasL, Fas and APO2 (apoptin 2) ligands to promote activation-induced cell death that may be required for immune responses (65).

To date, the role of MSC-derived exosomes (MSC-Exos) in immunomodulation is a popular research topic. MSC-Exos have been shown to influence the activities of B cells and T cells, stimulate angiogenesis and regulate cell apoptosis and inflammation processes by carrying various immune regulatory factors (cytokines, chemokines and growth factors) (66). For example, studies have shown that MSC-Exos can induce T cell apoptosis through FasL or activate the PD-L1 pathway to inhibit T cell activation, leading to immune tolerance and inducing the differentiation of Th1 cells into Th2 cells, which then reduces the levels of proinflammatory cytokines, such as IL-1β, IL-6, IL-12, and TNF-α, and increases the levels of anti-inflammatory cytokines, such as IL-10 and TGF-β (67–69). In addition, MSC-Exos can also act on macrophages, promoting the polarization of M2 macrophages and the secretion of anti-inflammatory cytokines by macrophages, which helps maintain local immune and metabolic homeostasis (70).

**Figure 1:**
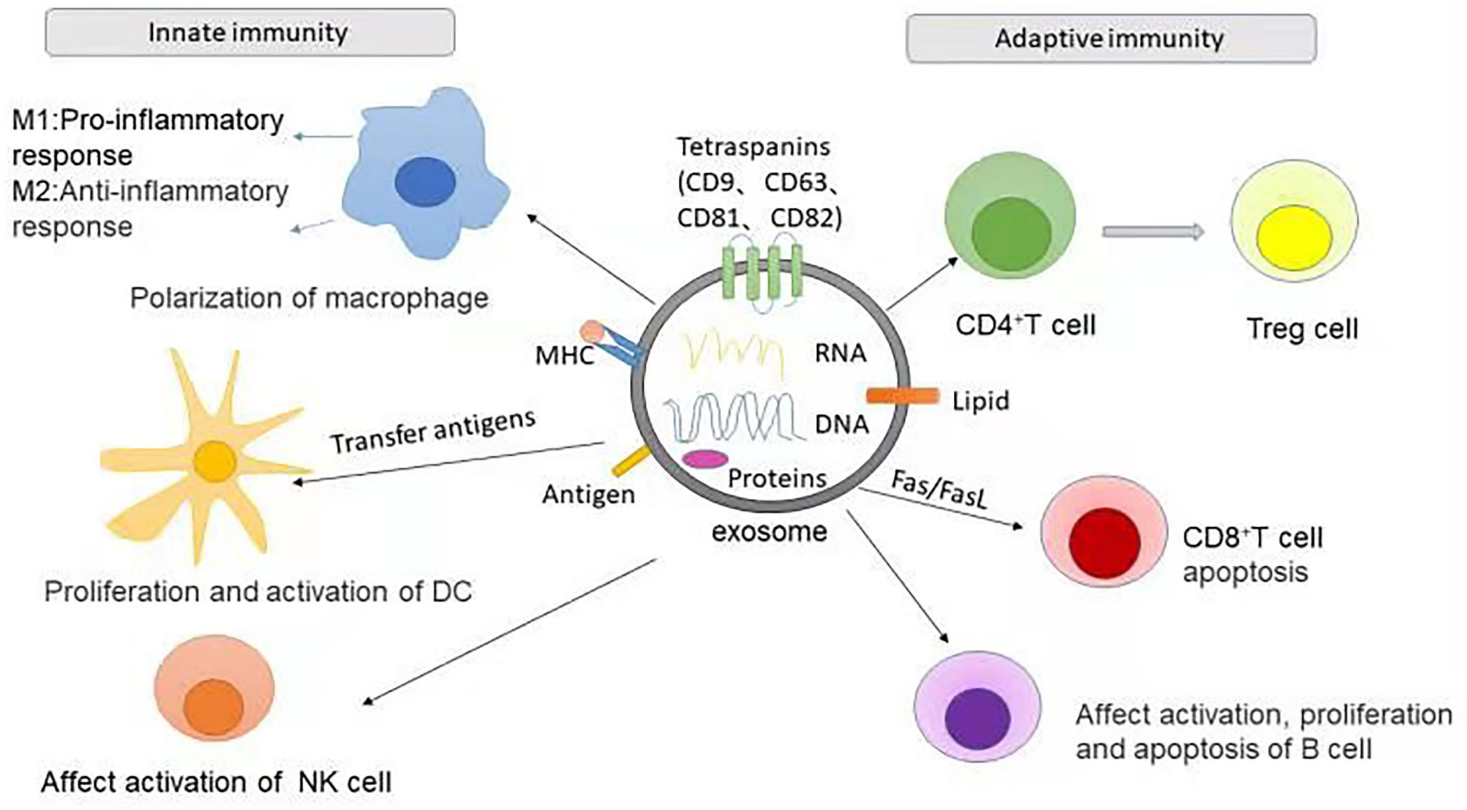
The role of exosomes in two types of immunity. In innate immunity, exosomes can promote the polarization of macrophages to M1 or M2 types, respectively leading to the promotion or inhibition of inflammatory response. The MHC complex and antigens carried by exosomes can present the antigen complex to DCs, thereby promoting the proliferation and activation of DCs. Exosomes can also activate NK cells or inhibit the cytotoxic activity of NK cells. The role of exosomes in adaptive immunity is to influence the activation, proliferation and apoptosis of T cells and B cells. For example, MSCs-derived exosomes can promote the differentiation of CD4+T cells into Treg cells and CD8+T apoptosis.

In addition, exosomes contribute significantly to the immunoregulation of Treg cells and Th17 cells. Human umbilical cord MSC-derived exosomes increase the proportion of CD4^+^CD25^+^Foxp3^+^ Treg cells and reduce the proportion of CD4^+^IL17A^+^ T cells (Th17 cells) (71). Moreover, it has been reported that (45) exosome like granules of thymocytes can induce the differentiation of CD4^+^CD25^-^ T cells into CD4^+^CD25^+^Foxp3^+^ Treg cells, which contributes to maintaining the immune tolerance of peripheral tissues. In summary, exosomes can affect the differentiation and function of Th cells (T helper cells) and Treg cells, and serve as intercellular mediators that regulate several types of immune responses (**Figure 1**).

## Exosomes and AITDs

### Exosomes and HT

HT, also known as chronic lymphocytic thyroiditis, is the most common autoimmune thyroid disease, accounting for approximately 22.5% of thyroid diseases (72). It is characterized by infiltration of thyroid-specific T lymphocytes and other immune cells, goiter enlargement and fibrosis, destruction of thyroid cells, and eventually hypothyroidism (73). A few HT patients have no goiter, and approximately 50% of patients have clinical hypothyroidism. The characteristic autoantibodies of HT mainly include TPOAb and TGAb (74), which have the function of fixing complement, causing cytotoxicity and damaging thyrocytes. In addition, HT patients may also have thyroid stimulating blocking antibodies (TSBAbs) (74), which can promote thyroid atrophy and hypofunction. In HT patients, Th1 cells are the dominant infiltrating lymphocytes, which release cytokines (IFN-γ, IL-2, and TNF-α) under the stimulation of thyroid autoantigen, and the latter stimulates the expression of Fas on the surface of the thyroid cells, thereby accelerating the apoptosis of the thyroid cells (75). Exosomes from human B cell-derived lymphoblastoid cell lines highly express MHC II and FasL, which can induce the apoptosis of CD4^+^ T cells through the interaction of FasL and Fas (40, 76, 77). Therefore, exosomes could be associated with cell apoptosis and participate in several pathological processes related to HT.

Exosomal surface protein markers such as HSP60 and HSP70 can mediate immunomodulatory effects and immune responses (78, 79). HSP60 can bind a variety of receptors present on the surface of immune cells, such as TLRs, CD40, and CD9, leading to antigen cross-presentation, T cell cross-priming, and immune response (78, 80). Cui et al. (81) detected serum exosomes of 40 HT patients and 40 healthy controls, and concluded that exosomes from HT patients (HT-Exos) highly expressed the inflammatory factor HSP60, which was positively correlated with serum TPOAb and TgAb. After coculture with healthy human DCs, HT-Exos derived HSP60 was spatially closely arranged and more fully bound to TLR2 on DCs compared with intracellular HSP60. Similarly, another study found that the level of HSP60 in thyroid follicular cell-derived exosomes stimulated by IFN-γ was significantly higher than that in normal thyrocyte-derived exosomes. Interestingly, the structure of HSP60 is highly homologous with the structures of TPO and TG, and mediates the disruption of thyrocytes through antigen-antibody reactions in HT (82). Therefore, there is a hypothesis that exosomal HSP60 may serve as an antigen to mediate immunological functions in HT pathogenesis. However, further studies are required to identify the mechanisms of exosomal HSP60-mediated regulation of HT pathogenesis.

Current studies have demonstrated that Th1/Treg, Th2/Treg and Th17/Treg cells in HT patients are out of balance at the transcription factor level, manifesting as the upregulation of Th1, Th2 and Th17 cells, and the downregulation of Treg cells (83–85). In this regard, exosomes from HT patients cocultured with peripheral blood mononuclear cells (PBMCs) of healthy humans increased the percentage of CD4^+^ IFN-γ^+^ Th1 cells and CD4^+^IL-17A^+^ Th17A cells in PBMCs, and decreased the percentage of CD4^+^CD25^+^Foxp3^+^ Treg cells in PBMCs (81). Furthermore, this study also found that exosomes from HT patients cocultured with DCs bind to TLR2/3 of DCs, and activate DCs through the TLR2/myeloid differentiation factor 88 (MyD88)/NF-κB and TLR3/TRIF/NF-κB pathways, thus leading to an imbalance in the differentiation of CD4^+^ T lymphocytes. In conclusion, these findings support a possible role for exosomes in HT pathogenesis: the levels and composition of circulating exosomes in HT patients could induce T cell imbalance.

As the most powerful professional APCs and CD4^+^ T lymphocytes, DCs are the main cells involved in the inflammatory response, and they participate in the pathogenesis of HT (86). Research has revealed that APC-derived exosomes play a role in antigen presentation and T lymphocyte activation. For example, mature DC-derived exosomes have been shown to induce the proliferation of CD4^+^ T cells in an antigen-specific manner (87), but the role of exosomes derived from nonprofessional APCs (such as thyroid follicular cells) in these processes is rarely described. Another study by Cui et al. (88) found that thyroid follicular cell-derived exosomes stimulated by IFN-γ (IFN-γ-Exos) cocultured with DCs increased the expression of costimulators CD40 and CD80 and the mature marker CD83 on DCs, and increased the gene expression levels of inflammatory cytokines such as IL-6 and TNF. Furthermore, after culturing DCs, IFN-γ-Exos cocultured with CD4^+^ T lymphocytes from healthy humans significantly increased the mRNA expression levels of IFN- γ, IL-17A, IL-22, IL-4, IL-10 and TGF-β1 in CD4^+^ T cells. All of the evidence suggests that exosomes could activate DCs to further cause an imbalance in cytokine expression and the secretion of CD4^+^ T cells, which may lead to the formation of thyroid tissue and a systemic inflammatory environment. These inflammatory microenvironments can further promote the release of “abnormal exosomes” from thyrocytes *in vivo*, forming a positive feedback loop involved in HT pathogenesis. Additionally, another study observed that the exosome inhibitor GW4869 blocked the activation of DCs by thyroid follicular cells after inhibiting IFN-γ-Exos, suggesting that exosomes play a key role in antigen presentation and the inflammatory response. GW4869 is a neutral sphingomyelinase inhibitor with cell permeability and selectivity, and it is basically recognized as an exosome inhibitor. It can inhibit the secretion of exosomes *in vivo* or *in vitro*, which has been reported in tumors, inflammation and infection (89–92). These studies indicate that intervention in exosome secretion may have a therapeutic effect on inflammation and autoimmune diseases. Together, these results suggest a potential role of thyroid follicular cell-derived exosomes in regulating T cell differentiation imbalance in the occurrence and development of HT, but the specific mechanism remains to be further clarified.

### Exosomes and GD

GD, namely, diffuse toxic goiter, is an organ-specific autoimmune disease with abnormal thyroid hormone synthesis, accounting for more than 85% of patients with hyperthyroidism, with a high incidence in women aged 40-60 years old (93). GD mainly manifests as thyrotoxicosis, diffuse goiters and Graves’ ophthalmopathy (GO) (94). Abnormal infiltration of T lymphocytes and production of the autoantibody TRAb are the pathophysiological basis of GD (95). The imbalance of Th1/Th2 cells has become a research focus of GD in recent years. It is generally believed that the inflammatory response mediated by Th1 cells is dominant in the active phase of GD, while Th2 cells mainly play a role in the inactive phase of GD (96). In GD, the recruitment of Th1 lymphocytes leads to increased production of IFN-γ and TNF-α, which stimulates thyroid cells to secrete Th1 chemokines, promoting the autoimmune process. A study (97) investigated the role of IL-21 in the regulation of Th17/Treg cells in 28 newly diagnosed GD patients, 27 remission GD (EGD) patients and 24 healthy subjects and found that IL-21 could stimulate the differentiation of CD4^+^ T cells into Th17 cells, reduce the differentiation of Treg cells, and contribute to the activation of the downstream immune response and the pathogenesis of GD.

A number of studies have implicated that IGF-1R and TSHR (thyrotropin receptor) are involved in GD pathogenesis and have a direct interaction in thyroid related eye diseases. Reports from Huang et al. (98) demonstrated that IGF-1R, TPO, TSHR and HSP60 existed in serum exosomes from GD patients and healthy controls. In particular, the levels of IGF-1R and TPO in the serum exosomes of GD patients and active GO patients without hormone shock therapy were significantly higher than those in healthy controls. Further correlation analysis showed that IGF-1R was significantly positively correlated with TRAb levels, suggesting that exosomes may present antigens and carry autoantigens (IGF-1R) in GD pathogenesis. However, further study is required to identify why circulating exosomes are specifically recognized as autoantigens in GD.

Additionally, Rossi and his colleagues observed that the formation and transfer of exosomes from thyroid follicular cells induced by dichlorodiphenyltrichloroethane (DDT) contained thyrotropin-releasing hormone, which could directly stimulate TSHR to binding to autoantibodies, leading to GD development (99). Interestingly, Edo et al. (100) provided evidence that TSHR is detected in exosomes secreted by normal thyroid follicular cell lines and cancerous thyroid cell lines. To examine the function of TSHR exosomes in GD, HEK (human embryonic kidney)/TSHR cells were generated as a model for thyroid follicular epithelial cells in GD where TSHR is upregulated. This study demonstrated that TSHR exosomes isolated from HEK/TSHR cells may exert a decoy effect by binding to and sequestering autoantibodies, thereby ameliorating autoantibody-mediated activation of thyroid function in a GD model. These results seem to contradict each other; therefore, it is necessary to further study the function of TSHR in exosomes in GD.

Circulating exosomes from GD patients were observed to be immunologically active and were capable of inducing the production of inflammatory cytokines from healthy PBMCs. Hiratsuka et al. (101) isolated serum exosomes from patients with refractory GD, and these exosomes stimulated the mRNA expression of inflammatory cytokines such as IL-1, IL-6 and TNF-α in PBMCs, thereby activating the immune response over that found in GD patients in remission or healthy controls. Similarly, Huang et al. (98) uncovered that coculture of serum exosomes from GD patients and healthy human PBMCs promoted the increased expression of CD11c^+^TLR2^+^ DCs and CD11c^+^TLR3^+^ DCs in PBMCs as well as an increase of IL-6 and IL-1β in the supernatant. These results suggest that exosomes from GD patients are able to promote the inflammatory response, favoring the pathogenesis and development of GD. To further investigate the specific mechanism of exosomes in promoting the inflammatory response, Cui et al. (102)recently analyzed serum exosomes from 26 healthy controls (HC-Exos), 26 GD patients (GD-Exos) and 7 Graves ophthalmopathy patients (GO-Exos). In this study, after coculture of healthy human PBMCs with GD-Exos or HC-Exos for 24 hours, GD-Exos could bind to TLR2/3 and increase the percentage of CD11c^+^TLR2^+^ DCs and CD11c^+^TLR3^+^ DCs in PBMCs; therefore, GD-Exos may mediate their role by acting on TLRs. Furthermore, GD-Exos also significantly increased the protein expression of MyD88, TRIF and p-P65 in PBMCs, as well as the levels of IL-6 and IL-1β in the medium. It is therefore possible that GD-Exos might participate in GD pathogenesis by binding to TLR2/3 to activate the NF-κB signaling pathway and induce an inflammatory response. While the mechanisms have not been fully revealed, these observations provide clues that exosomes can promote the inflammatory response in GD.

In addition to synergies between immune factors and environmental stimulation, epigenetics also plays an important role in the pathogenesis of GD, including DNA demethylation, histone modification and noncoding RNAs(ncRNAs) interference (103, 104). Intriguingly, the role of noncoding RNAs (including miRNAs, long noncoding RNAs, and circRNAs) in the etiopathogenesis of GD is increasingly attracting attention (105, 106). It has been revealed that nucleic acids contained within exosomes retain their function after exosomal vesicles direct transfer their contents into a recipient cell (107). Research has discovered differences in exosomal noncoding RNAs between healthy populations and GD patients at different stages. Hiratsuka et al. (101) indicated that plasma exosomes from GD patients carry upregulated or downregulated miRNA levels and are associated with disease progression. In this study, compared with intractable GD patients, the levels of miR-23b-5p and miR-92a-3p in serum exosomes of GD patients in remission were significantly increased, while the levels of let-7g-3p and miR-339-5p were significantly decreased. However, the molecular mechanisms of these miRNAs related to GD have not yet been fully elucidated. Other studies have discovered that miRNAs in serum exosomes of intractable GD patients can stimulate mRNA expression of IL-1β and TNF-α, which may be closely related to the pathogenesis and disease progression of GD. Additionally, Sun et al. (108) showed that plasma exosome circRNAs from GD patients were more than twice as upregulated or downregulated compared with healthy controls, with 15 circRNAs significantly differentially expressed, including the upregulation of 6 circRNAs and the downregulation of 9 circRNAs. This study further verified the significantly increased expression level of has-circRNA-000102 in the plasma exosomes of GD patients and revealed the potential pathways related to GD pathogenesis, namely, herpes simplex virus infection, influenza A signaling and IFN-β signaling. These observations imply the potential roles of exosome-derived noncoding RNAs as significant regulators and biomarkers for diagnosing GD. However, more studies are required to investigate exosomal RNAs and their significance in the pathophysiology and diagnosis of GD.

Thyroid ophthalmopathy (TED) is the most common extrathyroid manifestation of AITD. It includes GO, which in severe cases can result in visual field loss due to corneal rupture or compression optic neuropathy (109). Han et al. (110) isolated tear exosomes from GD patients with TED, and found that the tear exosomes of TED patients were 2.3 times higher than those of healthy controls, and highly expressed vitamin D binding protein (VDBP), C-reactive protein (CRP), chitinase 3-like 1 (CH3L1), matrix metalloproteinase-9 (MMP-9) and vascular adhesion molecule-1 (VCAM-1).

Matrix metalloproteinases play a key role in tissue remodeling in the process of fibrosis and inflammation. They are expressed at very low levels in normal tissues, but can be increased by the influence of inflammatory cytokines, intercellular interactions, hormones, and growth factors (111). Tear derived exosomes can trigger orbital fibroblasts to release the inflammatory cytokines, IL-6, IL-8 and monocyte chemoattractant protein-1 (MCP1) *in vitro*. These findings suggest that an increased abundance of specific proteins in exosomes can also activate inflammatory responses through orbital fibroblasts (the target cells in TED). In general, increased levels of MMP-9 in tear exosomes could promote orbital tissue remodeling and fibrosis, which contributes to the occurrence and development of TED.

## Conclusions

As a newly discovered biological carrier, exosomes contain various proteins, nucleic acids, lipids and other biological information that can regulate the docking and membrane fusion between exosomes and target cells, thus affecting the occurrence and progression of many diseases (112). Recent studies have highlighted the ability of exosomes to regulate immune responses and their great potential as biomarkers for detecting autoimmune diseases. As the most common organ-specific autoimmune disease, AITDs are caused by a complex interaction of genetic and environmental factors. Autoimmune intolerance and T lymphocyte imbalance are important factors in the pathogenesis of AITDs. It is now known that exosomes can affect the balance of Treg and Th17 cell differentiation and induce inflammatory responses, leading to the breakdown of autoimmune tolerance in AITDs. In addition, exosomes and their cargos from AITD patients are reported to be upregulated and participate in numerous biological processes as well as in AITD pathogenesis. Based on the current findings, the potential application of exosomes as diagnostic biomarkers and therapeutics for AITDs is a future opportunity. However, the basic and applied research on exosomes is still in its early stage, and the potential mechanism in AITDs has not been fully clarified. Consequently, continued research is required to elucidate the exact mechanism of exosomes in AITDs, thus contributing to a deeper understanding of disease diagnosis and prognosis.

## Author Contributions

JZ drafted the manuscript. HP and YL designed the study and revised the manuscript. All authors contributed to the article and approved the submitted version.

## Funding

This work was supported by National Natural Science Foundation of China (Grant No. 81800698), Zhenjiang Science and Technology Planning Project (Grant No. SH2021026, SH2021059).

## Conflict of Interest

The authors declare that the research was conducted in the absence of any commercial or financial relationships that could be construed as a potential conflict of interest.

## Publisher’s Note

All claims expressed in this article are solely those of the authors and do not necessarily represent those of their affiliated organizations, or those of the publisher, the editors and the reviewers. Any product that may be evaluated in this article, or claim that may be made by its manufacturer, is not guaranteed or endorsed by the publisher.
